# Age-group differences between young and middle-aged adults in spatiotemporal EEG dynamics revealed by instantaneous frequency microstate analysis

**DOI:** 10.3389/fnagi.2026.1707228

**Published:** 2026-03-26

**Authors:** Sou Nobukawa, Takashi Ikeda, Mitsuru Kikuchi, Tetsuya Takahashi

**Affiliations:** 1 Department of Computer Science, Chiba Institute of Technology, Narashino, Chiba, Japan; 2 Graduate School of Information and Computer Science, Chiba Institute of Technology, Narashino, Chiba, Japan; 3 Research Center for Mathematical Engineering, Chiba Institute of Technology, Narashino, Chiba, Japan; 4 Department of Preventive Intervention for Psychiatric Disorders, National Institute of Mental Health, National Center of Neurology and Psychiatry, Tokyo, Japan; 5 Research Center for Child Mental Development, Kanazawa University, Kanazawa, Ishikawa, Japan; 6 Department of Psychiatry and Behavioral Science, Kanazawa University, Kanazawa, Ishikawa, Japan; 7 Department of Neuropsychiatry, University of Fukui, Yoshida, Fukui, Japan; 8 Uozu Shinkei Sanatorium, Uozu, Toyama, Japan

**Keywords:** aging, aperiodic activity, electroencephalography (EEG), hidden Markov model (HMM), instantaneous frequency (IF), microstates

## Abstract

**Introduction:**

The human brain exhibits complex functions that emerge from interactions among spatially distributed neural regions. Electroencephalography (EEG) microstate analysis has been widely adopted to capture transient topographies reflecting large-scale network dynamics; moreover, it has been linked to cognitive functions, intrinsic brain networks, and neuropsychiatric disorders. Building on this framework, we recently proposed a novel approach based on instantaneous frequency (IF), defined as the temporal derivative of the instantaneous phase, which characterizes microstates in a dimension distinct from that of conventional amplitude-based microstates by explicitly capturing the phase leading and lagging. Although IF microstates have shown promise in characterizing the pathology and cognitive decline in Alzheimer's, their relevance to normal aging has not been investigated. This study aimed to identify age-group differences in large-scale EEG-dynamic properties using IF microstates.

**Methods:**

We recorded resting-state EEG with eyes closed from 29 younger and 18 middle-aged healthy adults. IF time series were extracted from sensor-level EEG signals in the theta and alpha bands. The IF microstates were identified using a hidden Markov model to ensure temporal continuity in state segmentation. Subsequently, we evaluated the sensor-level spatial distributions, mean dwell times, occupancy, and transition probabilities of the IF microstates and assessed age-group differences using appropriate statistical tests with false discovery rate correction.

**Results:**

We identified several IF microstates characterized by frontal IF delay and occipital IF lead, as well as microstates deviating from these patterns. Group comparisons revealed age-group differences in dynamic properties; in the middle-aged group, mean dwell times increased in some states and decreased in others, while occupancy and transition probabilities also exhibited significant changes.

**Discussion:**

IF microstate analysis provides a novel and informative perspective on age-group differences in spatiotemporal EEG dynamics. This approach, which is distinct from conventional amplitude-based microstates, may be useful for understanding healthy aging neural mechanisms.

## 1 Introduction

The human brain exhibits diverse and complex functions emerging from the hierarchical interactions between spatially distributed neural regions (Sporns and Betzel, 2016; Battiston et al., 2020; Thiebaut de Schotten and Forkel, 2022; Nava et al., 2024). Specifically, the dynamics of large-scale neural networks—arising from reciprocal influences among local processing units—are crucial in supporting higher-order cognitive functions by regulating information transmission, integration, and coordination. Such network dynamics can be characterized by multiple indices, including functional connectivity and its topological properties; phase–amplitude coupling (PAC), which reflects cross-frequency interactions; and dynamic functional connectivity, which captures time-varying fluctuations (Aru et al., 2015; Hutchison et al., 2013; Esghaei et al., 2022). Therefore, evaluating whole-brain activity patterns, rather than focusing solely on local activity, is important to gain deeper insight into brain states (Raut et al., 2021).

Among the methods for capturing the dynamics of large-scale neural networks, electroencephalography (EEG) microstate analysis has been widely adopted in a wide range of cortical brain activities (Lehmann et al., 1987; Lehmann, 1971). By segmenting EEG signals into temporally stable topographical patterns, microstate analysis provides a quantitative description of neural states. Previous studies have associated microstates with various cognitive functions (Michel and Koenig, 2018), large-scale brain networks such as the default mode network, and neuropsychiatric disorders, including schizophrenia and dementia (Van de Ville et al., 2010; Musso et al., 2010; Khanna et al., 2015; Rajkumar et al., 2021; Zhang et al., 2021). Recently, microstates have been associated with dynamic functional connectivity, suggesting their utility in understanding large-scale network dynamics (Hao et al., 2022; Guan et al., 2022; Yan et al., 2023). Novel approaches have also been proposed for defining microstates directly from network-level features (Dimitriadis et al., 2013, 2015).

Recent EEG studies, including those incorporating microstate analyses, have increasingly emphasized the importance of the instantaneous phase. Specifically, the theta phase information is a key marker for large-scale integration and communication across cortical regions (Tobe et al., 2023). Moreover, the theta phase modulates the local gamma activity through PAC, providing a mechanism for cross-scale coordination (Esghaei et al., 2022). Additionally, temporal variations in alpha band phase differences between regions reflect the moment-to-moment reconfiguration of functional networks (Nobukawa et al., 2019). Therefore, phase-based metrics may be essential for understanding the dynamic aspects of brain networks. Building on this line of research, we recently proposed a novel microstate framework based on instantaneous frequency (IF)—defined as the temporal derivative of the instantaneous phase—of theta and alpha oscillations (Nobukawa et al., 2021). IF microstates capture spatial patterns similar to those of conventional amplitude-based microstates, while additionally encoding features of phase leading and phase lagging. We have demonstrated that the frequency and transition probabilities of IF microstates are significantly associated with the pathology and cognitive decline in Alzheimer's disease (Nobukawa et al., 2024), highlighting their potential as a complementary perspective on brain state dynamics. Nonetheless, to the best of our knowledge, IF microstate analysis has not yet been used to investigate age-related changes in healthy individuals. During normal aging, brain functions, including attention, memory, and executive functions, gradually decline (Birren and Fisher, 1995). In a super-aging society, characterizing the alterations in spatiotemporal neural dynamics associated with aging is crucial for understanding brain function and developing preventive strategies.

Thus, we hypothesized that IF microstate analysis would reveal novel aspects of age-group differences in spatiotemporal EEG patterns. To test this hypothesis, we aimed to identify IF microstates and evaluate their dynamic properties, including occupancy and state-transition probabilities, using EEG data from 29 younger and 18 middle-aged healthy adults (Nobukawa et al., 2019). To ensure temporal continuity in the state identification, we employed a hidden Markov model (HMM)–based clustering approach.

## 2 Materials and methods

### 2.1 Participants

**Figure 1:**
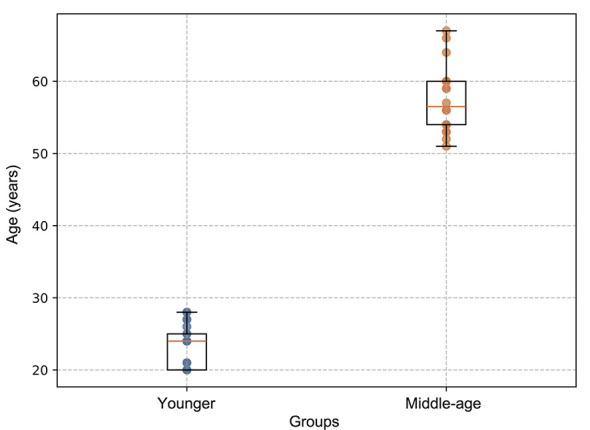
Age distribution of participants in the younger and middle-aged groups. Box plots show the median (central line), interquartile range (box), and range excluding outliers (whiskers), with individual participant data points overlaid.

We recruited 29 healthy young adults (14 males, 15 females; mean age 22.9 ± 2.7 years, range 20–28 years) and 18 healthy middle-aged adults (seven males, 11 females; mean age 57.5 ± 4.7 years, range 51–67 years) from Kanazawa University (Nobukawa et al., 2019). The age distribution of participants in the younger and middle-aged groups is shown in Figure 1. The assumed effect size (Cohen's *d* = 0.8) was informed by prior analyses conducted on the same dataset (Nobukawa et al., 2019; Ando et al., 2022), which yielded effect sizes of approximately η^2^ = 0.2–0.3 for related group comparisons in evaluations of EEG signal dynamics. Under a two-group framework, these values correspond to large effects (approximately *d*≈1.0–1.3). Based on this assumption, an a priori power analysis using a two-sided independent-samples *t*-test (α = 0.05, power = 0.80) indicated that a total sample size of approximately *N*≈52 (26 participants per group) would be required. The present sample size (*N* = 47) is close to this requirement. There was no significant difference in the sex distribution between the groups (χ^2^ = 0.39, *p* = 0.52). All participants were nonsmokers and were not on any medications. The exclusion criteria included a history of epilepsy, head injury, drug dependence, or other major medical or neurological disorders, as well as the presence of brain abnormalities detectable by routine clinical magnetic resonance imaging. In the middle-aged group, participants with mini-mental state examination scores below 27 were excluded. Years of education were also recorded for all participants. In the younger group, participants had either 14 years (*N* = 15) or 18 years of education (*N* = 8), corresponding to university-level education. In the middle-aged group, years of education ranged from 12 to 16 years, with the majority of participants having 14 years of education (*n* = 13), and smaller subsets having 12 years (*n* = 3) or 16 years (*n* = 2). All participants were fully informed about the experimental procedures. The study protocol was approved by the Ethics Committee of Kanazawa University, and written informed consent was obtained from all participants.

### 2.2 EEG acquisition

**Figure 2:**
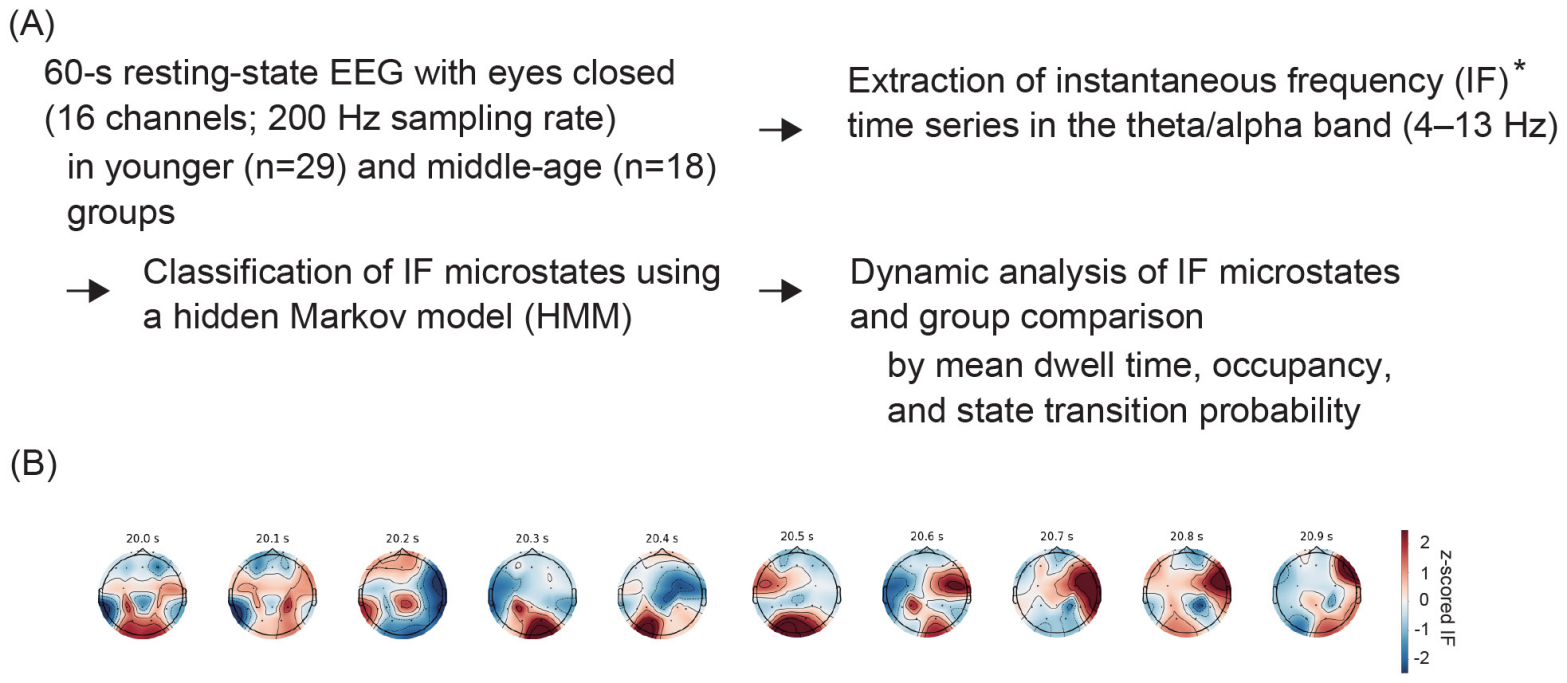
**(A)** Conceptual overview of preprocessing, instantaneous frequency (IF) derivation, and IF microstate estimation process. **(B)** Scalp topographic maps of spatially *z*-scored IF values, displayed as a typical example of a time series (20–25 s, every 0.1 s) in the process indicated by the asterisk.

EEG data were collected in a shielded, sound-attenuated, darkened recording room. Sixteen scalp electrodes (Fp1, Fp2, F3, Fz, F4, F7, F8, C3, C4, P3, Pz, P4, T5, T6, O1, and O2) were placed according to the international 10–20 system using a Nihon Kohden EEG-4518 amplifier (Nihon Kohden Corporation, Tokyo, Japan) with linked earlobe electrodes as the reference. Bipolar electrooculography was simultaneously performed to monitor eye movement. The signals were sampled at 200 Hz (time constant 0.3 s) and bandpass-filtered between 1.5 and 60 Hz. The participants were instructed to rest seated with their eyes closed for 10–15 min, and their vigilance level was monitored by video recording. No visual stimuli were presented during the recording, and participants were not seated in front of a monitor. Epochs demonstrating signs of drowsiness or sleep were excluded, as were segments contaminated with muscle artifacts, blinks, or eye movements, which were removed by visual inspection. For each participant, a continuous artifact-free segment of 60 s (12,000 samples) was extracted. This data segment was further bandpass-filtered between 4 Hz and 13 Hz (theta–alpha range) (Nobukawa et al., 2024). An overview of the subsequent IF time series extraction and microstate estimation processes is shown in Figure 2A.

### 2.3 IF microstates

#### 2.3.1 Extraction of instantaneous frequency

Here, brain states were defined based on the IF spatial distribution. First, EEG signals were bandpass-filtered between 4 and 13 Hz to extract both theta (4 − 8 Hz) and alpha (8 − 13 Hz) bands (Nobukawa et al., 2024), which are known to be associated with large-scale neural interactions. To minimize the edge artifacts introduced by filtering, the first and last 5 s of each epoch were removed. To examine the robustness of the IF microstate structure under a narrower frequency condition, we additionally performed the same analysis restricted to the alpha band (8 − 13 Hz), and the results are provided in the Supplementary material.

For each channel *i*, the analytic signal *a*_*i*_(*t*) was obtained by applying a Hilbert transform to the bandpass-filtered signal *x*_*i*_(*t*). The wrapped instantaneous phase was then defined as:


$$\begin{array}{l} \phi_{i}\left(t\right)=\arg\left(a_{i}\left(t\right)\right),\;-\pi \leq \phi_{i}\left(t\right)\leq \pi . \end{array}$$ (1)

To compute IF, the phase was first unwrapped:


$$\begin{array}{l} \text{unwrap}\left[\phi_{i}\left(t\right)\right]=\phi_{i}\left(t\right)+2\pi k\left(t\right), \end{array}$$ (2)

where *k*(*t*)∈ℤ is an integer that corrects phase discontinuities. The instantaneous frequency was calculated as the temporal derivative of the unwrapped phase.


$$\begin{array}{l} \text{I}{\text{F}}_{i}\left(t\right)=\frac{1}{2\pi }\frac{d\text{unwrap}\left[\phi_{i}\left(t\right)\right]}{dt}. \end{array}$$ (3)

To reduce the influence of phase slips and high-frequency noise, IF_*i*_(*t*) was smoothed using a 100-ms median filter, following the methodology from a previous study (Nobukawa et al., 2019).

#### 2.3.2 Estimation of IF microstates

To extract spatial features, the mean across electrodes was subtracted from IF_*i*_(*t*) at each time point, and the resulting values were *z*-score normalized. These normalized deviations were concatenated across the 16 channels to form a 16-dimensional time series at each time point. A representative example of scalp topographic maps of the spatially normalized IF values is shown in Figure 2B. The time series from all participants in both age groups were concatenated, and clustering was performed using *k*-means with Euclidean distance. Subsequently, the resulting cluster centroids were used as initial states for HMM-based estimation of IF microstates. Each cluster centroid represents a dynamic EEG state characterized by a distinct IF spatial pattern, referred to here as an IF microstate.

The HMM employed in this study assumes a first-order Markov property, such that the probability of the current hidden state depends only on the immediately preceding state. Under this assumption, temporal continuity of IF microstates is modeled through probabilistic state transitions over time. To ensure direct comparability of IF microstates across age groups, the HMM was initialized using common state definitions obtained from *k*-means clustering applied to the combined dataset across both groups (Nobukawa et al., 2024), rather than fitting separate models for each group. This procedure prevents group-specific state reorganization and ensures consistent state identity across groups. Under these shared model parameters, individual state sequences were inferred separately for each participant, from which dwell times and transition probabilities were derived at the individual level for subsequent group comparisons.

To determine the optimal number of IF microstates, we computed the Akaike information criterion (AIC) and Bayesian information criterion (BIC) for each candidate model. The optimal number of states was selected based on the elbow point identified from changes in the slopes of these criteria.

### 2.4 Evaluation indexes

To quantify the temporal dynamics of each IF microstate, we computed two indices: the mean dwell time and occurrence. The mean dwell time (s) was defined as the average duration of individual microstate episodes *i*:


$$\begin{array}{l} D_{i}=\frac{1}{N_{i}}\overset{N_{i}}{\underset{e=1}{\sum }}d_{i,e}. \end{array}$$ (4)

where *N*_*i*_ is the total number of times microstate *i* appears and *d*_*i, e*_ denotes the duration of the *e*-th episode of microstate *i*. Occurrence was defined as


$$\begin{array}{l} f_{i}=\frac{\sum_{e=1}^{N_{i}}d_{i,e}}{T}, \end{array}$$ (5)

where *T* denotes the total duration of the evaluation.

Furthermore, to evaluate the transitions between the IF microstates, we computed the state transition probability for each ordered pair of microstates (*i*→*j*). The transition probability from microstate *i* to *j* was defined as follows:


$$\begin{array}{l} P\left(i\to j\right)=\frac{N_{i\to j}}{\underset{k}{\sum }N_{i\to k}}, \end{array}$$ (6)

where *N*_*i*→*j*_ is the number of times a state *i* is immediately followed by a state *j*, and the denominator sums the transitions from *i* to all the possible subsequent states *k*.

### 2.5 Statistical analysis

To investigate the group differences in microstate dynamics, we evaluated two emergence-related metrics (occurrence and mean dwell time), as well as state transition probabilities. For each metric, the data were first log-transformed to reduce skewness and mitigate outlier effects. Log-transformed data normality was tested using the D'Agostino–Pearson test. If both groups satisfied the normality assumption, group comparisons between younger and middle-aged adults were performed using two-sample *t*-tests. If the normality assumption was violated in either group, the non-parametric Mann–Whitney *U* test was applied. Multiple-comparison control was performed using the false discovery rate (FDR) procedure with a significance threshold of *q* < 0.05. For emergence-related metrics, four *p*-values were corrected within each matrix. For state transition probabilities, FDR correction was applied across the full *k*×*k* set of *p*-values corresponding to all possible transitions among *k* microstates.

## 3 Results

**Figure 3:**
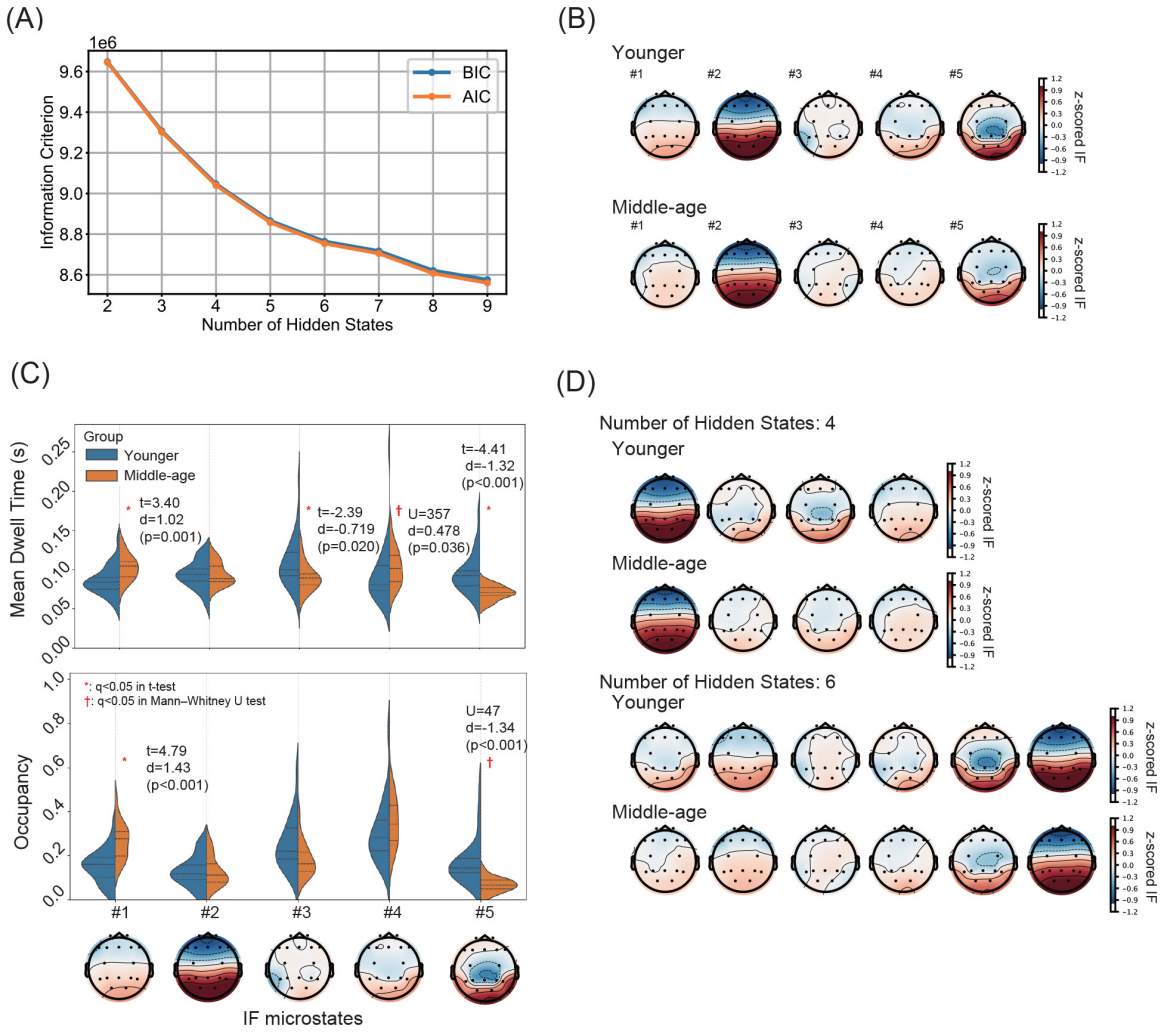
**(A)** Dependence of the number of IF microstates on the Akaike information criterion (AIC) and the Bayesian information criterion (BIC). An elbow point appears when the number of IF microstates is set to five. **(B)** Group-averaged spatial distributions of *z*-scored IF, estimated from multichannel IF time series, for each IF microstate in the younger and middle-aged groups. A pattern characterized by relatively faster occipital IF is observed in several IF microstates (e.g., #1, #2, #4, and #5), providing a functional interpretation of the state labels. **(C)** Violin plots of mean dwell times (top) and occupancy (bottom) for each IF microstate in the younger and middle-aged groups. The black solid line and dashed line within the distributions indicate the group mean and standard deviation, respectively. In the middle-aged group, mean dwell times exhibit marked increases and decreases in states #1 and #4 and #3 and #5, respectively. Occupancy demonstrates a marked increase and decrease in states #1 and #5, respectively. **(D)** Spatial distributions of *z*-scored IF microstates for (top) four and (bottom) six hidden states. In both cases, IF microstates characterized by frontal IF delay and occipital IF lead, as well as additional microstates with distinct spatial distributions, were observed, consistent with the optimal solution obtained for five hidden states, indicating that the overall patterns were robust to the choice of the number of hidden states.

Figure 3 displays the IF microstate analysis results. First, the dependence of the number of IF microstates on AIC and BIC was examined (Figure 3A). An elbow point was observed when the number of IF microstates was set to five, suggesting an optimal size for hidden states. Next, group-averaged spatial distributions of *z*-scored IF, estimated from multichannel IF time series, are presented for each IF microstate in the younger and middle-aged groups (Figure 3B). In one of the microstates, a pattern characterized by faster occipital IF was evident. Based on these microstates, violin plots of mean dwell times and occupancy for each IF microstate are shown in Figure 3C. All reported effects survived FDR correction (*q* < 0.05), and raw *p*-values are reported in the text for completeness. In the middle-aged group, the mean dwell times markedly increased in states #1 and #4 and decreased in states #3 and #5. Additionally, occupancy markedly increased in state #1 and decreased in state #5. Specifically, dwell time for IF microstate #1 was significantly longer in the middle-aged group than in the younger group (middle-aged: 0.103 ± 0.016 (group mean and standard deviation) s; younger: 0.086 ± 0.018 s, *p* = 0.001, Cohen's *d* = 1.02). In contrast, IF microstate #3 exhibited a shorter dwell time in the middle-aged group compared with the younger group (middle-aged: 0.090 ± 0.017 s; younger: 0.109 ± 0.033 s; *p* = 0.020, Cohen's *d* = −0.719). In addition, IF microstate #4 showed a modest but significant increase in dwell time in the middle-aged group (middle-aged: 0.103 ± 0.023 s; younger: 0.093 ± 0.037 s; *p* = 0.036, Cohen's *d* = 0.478). The largest group difference was observed for IF microstate #5, where dwell time was markedly shorter in the middle-aged group (middle-aged: 0.070 ± 0.009 s; younger: 0.094 ± 0.025 s; *p* < 0.001, Cohen's *d* = −1.32). Occupancy of IF microstate #1 was substantially higher in the middle-aged group than in the younger group (middle-aged: 0.265 ± 0.081; younger: 0.154 ± 0.076; *p* < 0.001, Cohen's *d* = 1.43). Conversely, IF microstate #5 showed markedly reduced occupancy in the middle-aged group (middle-aged: 0.068 ± 0.032; younger: 0.170 ± 0.101; *p* < 0.001, Cohen's *d* = −1.34). Furthermore, to evaluate alternative choices for the number of hidden states, the spatial distributions of *z*-scored IF microstates for the four and six hidden states are presented in Figure 3D. In both cases, IF microstates characterized by frontal IF delays and occipital IF leads, as well as additional microstates with distinct spatial distributions, were observed. These findings are consistent with the optimal solution obtained for the five hidden states, indicating that the overall patterns are robust to the choice of the number of hidden states.

**Figure 4:**
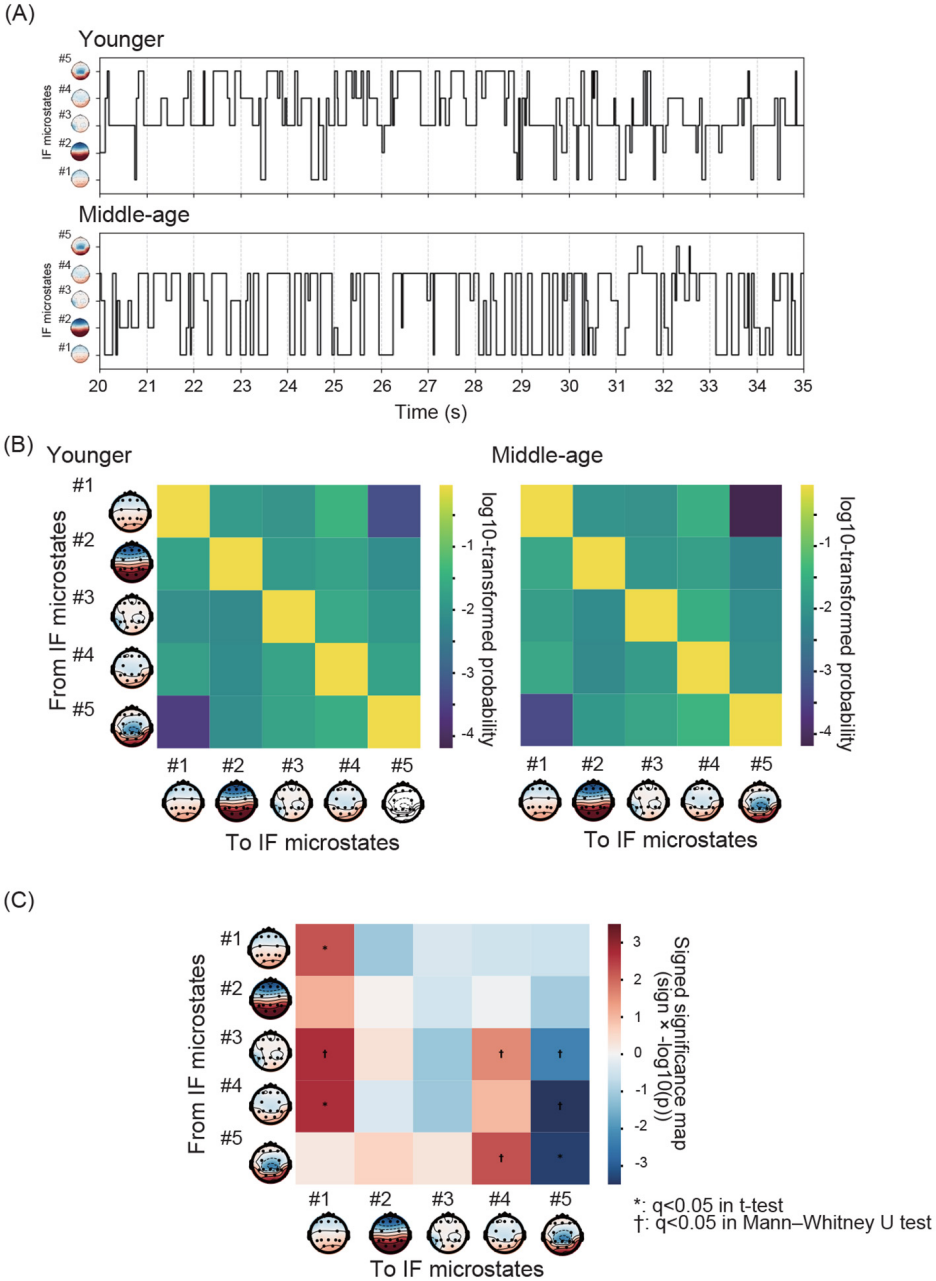
**(A)** Representative example of IF microstate transitions in a younger and a middle-aged participant. **(B)** Mean of state transition probabilities within each group (younger and middle-aged groups). **(C)** Signed significance score maps of state transition probabilities between IF microstates comparing younger and middle-aged groups. Positive (negative) values indicate higher transition probabilities in the middle-aged (younger) group. The color scale shows −log_10_(*p*), and statistical significance was assessed at *q* < 0.05 (FDR corrected). Several transitions showed significant group differences according to this criterion, including #1 → #1 (Cohen's *d* = 1.01), #3 → #1 (*d* = 1.17), #3 → #4 (*d* = 0.60), #3 → #5 (*d* = −0.86), #4 → #1 (*d* = 1.17), #4 → #5 (*d* = −1.29), #5 → #4 (*d* = 1.01), and #5 → #5 (*d* = −1.39). Positive (negative) values of Cohen's *d* indicate higher transition probabilities in the middle-aged (younger) group.

In Figure 4A, representative examples of IF microstate transitions in the younger and middle-aged participants are displayed. Based on these transitions, we first compared the mean state transition probabilities between IF microstates in the younger and middle-aged groups (Figure 4B). To statistically evaluate group differences, we computed signed significance scores based on the sign of the group difference and −log_10_(*p*) values obtained from two-sample tests (Welch's *t* test or Mann–Whitney *U* test, depending on normality). The resulting significance maps (Figure 4C) revealed several transitions that differed significantly between the younger and middle-aged groups, with positive scores indicating higher transition probabilities in the middle-aged group (from #1 to #1, from #4 to #1, from #3 to #5, and from #4 to #5) and negative scores indicating lower values in the middle-aged group (from #3 to #1, from #3 to #4, from #5 to #4, and from #5 to #5). Several state transition probabilities exhibited significant age-group differences after FDR correction. Self-transition probability of IF microstate #1 was significantly higher in the middle-aged group than in the younger group (middle-aged: 0.950 ± 0.008; younger: 0.939 ± 0.012; *p* = 0.002, Cohen's *d* = 1.01). In contrast, self-transition probability of IF microstate #5 was significantly lower in the middle-aged group (middle-aged: 0.928 ± 0.010; younger: 0.944 ± 0.013; *p* < 0.001, Cohen's *d* = −1.39). Regarding cross-state transitions, transitions from IF microstate #3 to #1 occurred more frequently in the middle-aged group (middle-aged: 0.014 ± 0.006; younger: 0.008 ± 0.005; *p* < 0.001, Cohen's *d* = 1.17), whereas transitions from IF microstate #3 to #5 were reduced (middle-aged: 0.007 ± 0.003; younger: 0.012 ± 0.006; *p* = 0.001, Cohen's *d* = −0.86). Transitions from IF microstate #3 to #4 also showed a modest increase in the middle-aged group (middle-aged: 0.029 ± 0.007; younger: 0.023 ± 0.011; *p* = 0.008, Cohen's *d* = 0.60). Similarly, transitions from IF microstate #4 to #1 were more frequent in the middle-aged group (middle-aged: 0.022 ± 0.006; younger: 0.015 ± 0.005; *p* < 0.001, Cohen's *d* = 1.17), whereas transitions from IF microstate #4 to #5 were markedly reduced (middle-aged: 0.008 ± 0.004; younger: 0.018 ± 0.009; *p* < 0.001, Cohen's *d* = −1.29). Finally, transitions from IF microstate #5 to #4 were significantly increased in the middle-aged group (middle-aged: 0.039 ± 0.011; younger: 0.029 ± 0.010; *p* = 0.001, Cohen's d = 1.01).

## 4 Discussion

Here, we extracted IF time series in the alpha/theta bands from resting-state EEG with eyes closed in healthy younger and middle-aged adults and evaluated IF microstates using HMM. Several IF microstates were characterized by frontal IF delay with occipital IF lead, along with microstates deviating from this pattern. Furthermore, age-group differences were observed in mean dwell times, occupancy, and IF microstate transition probabilities.

The emergence of multiple IF microstates characterized by frontal delay and occipital lead property is related to the well-known phenomenon (Robinson et al., 2001, 2003; O'Connor and Robinson, 2004; Gray and Robinson, 2013; Chiang et al., 2011). The higher occipital alpha oscillation frequency compared with that of the frontal regions has been attributed to shorter corticothalamic conduction delays in the posterior regions (Robinson et al., 2003; O'Connor and Robinson, 2004). Therefore, the structural heterogeneity of the brain may give rise to IF microstates characterized by occipital lead.

Furthermore, we examined age-group differences in the occupancy and transition properties of IF microstates. Recent studies have highlighted the contribution of aperiodic components (previously treated as background or 1/*f* activity) to alpha-band EEG signals, which also change with age. Indeed, several reports have shown that apparent age-related differences in alpha-band measures are substantially reduced when these aperiodic components are explicitly controlled (Merkin et al., 2023; Cesnaite et al., 2023). Moreover, our previous work reported increased complexity of phase-difference time series in the alpha band, particularly in frontal regions, with aging (Nobukawa et al., 2019). Taken together, age-related alterations in alpha-band EEG activity—including changes in aperiodic components and phase-difference patterns—may contribute to the observed differences in IF microstate dynamics. Nevertheless, disentangling the relative contributions of periodic and aperiodic components will require future studies employing explicit spectral decomposition and control analyses.

The limitations of this study must be considered. First, it should be noted that the present study focused on a limited age range spanning young adulthood to early-to-mid adulthood. Therefore, the observed differences should not be interpreted as representing general aging effects across the full lifespan or late-stage aging. Rather, the findings reflect age-group differences within this specific developmental window. Future studies employing broader age ranges, including late adulthood, as well as larger-scale datasets will be necessary to validate the generalizability and robustness of the proposed IF microstate framework. Second, the present study employed a 16-channel EEG system, which inherently limits spatial resolution. Accordingly, the observed spatial patterns, such as frontal delay or occipital lead, should be interpreted at the sensor level rather than as precise cortical or source-localized effects. While the present results demonstrate robust phase-based temporal organization across electrode groups, future studies using high-density EEG and source-based analyses will be required to establish detailed cortical or network-level interpretations. Third, spectral features such as power spectrum, aperiodic slope, and alpha peak frequency were not explicitly controlled in the present analysis. As a result, the observed age-group differences in IF microstate dwell times and transition properties cannot be interpreted as independent age-related alterations in neural dynamics. Instead, these differences may reflect, at least in part, age-associated changes in underlying spectral and aperiodic components of alpha-band EEG activity. Future studies incorporating explicit spectral decomposition and control analyses will be required to disentangle the relative contributions of periodic and aperiodic components to IF microstate dynamics. Fourth, the resting-state EEG recording duration per participant was relatively short (60 seconds). Although the group-level HMM framework with shared state definitions facilitated reliable estimation of IF microstate dynamics for group-level comparisons, longer recordings will be required to assess the stability of dwell times and transition probabilities at the individual level and to further validate state dynamics across broader temporal scales. Fifth, the present study did not include cognitive or behavioral assessments. Therefore, the functional relevance of the observed IF microstate dynamics to cognition, memory, or executive function cannot be directly established. In addition, although years of education were recorded for all participants, their potential role in modulating age-group differences in IF microstate dynamics was not examined. Education may interact with age-related neural characteristics through mechanisms such as cognitive reserve; however, this possibility could not be evaluated in the present cross-sectional design and should be addressed in future studies with larger samples. While our previous study (Nobukawa et al., 2024) suggested that IF microstate transition properties may be related to cognitive decline in clinical populations, the present findings in healthy adults should be interpreted as methodological and descriptive. Future studies integrating standardized cognitive measures will be essential to clarify the translational relevance of IF microstate dynamics in aging.

## 5 Conclusion

In conclusion, by applying a HMM to IF microstate analysis, we evaluated age-group differences in sensor-level EEG dynamics between healthy younger and middle-aged adults. The identified IF microstates exhibited characteristic sensor-level patterns, including frontal delay and occipital lead, in middle-aged participants, particularly in their state occupancy and transition properties. These findings suggest that IF microstate analysis may provide a useful framework for characterizing age-group differences in EEG dynamics. Future work should extend the present approach to broader age ranges and larger datasets, and further examine how these IF microstate properties relate to aperiodic components and phase-difference patterns previously reported in the literature.

## Data Availability

The datasets presented in this article are not readily available because the informed consent did not include the declaration regarding publication of clinical data. Requests to access the datasets should be directed to Sou Nobukawa, nobukawa@it-chiba.jp.

## References

[B1] Ando M. Nobukawa S. Kikuchi M. Takahashi T. (2022). Alteration of neural network activity with aging focusing on temporal complexity and functional connectivity within electroencephalography. Front. Aging Neurosci. 14:793298. doi: 10.3389/fnagi.2022.793298 35185527 PMC8855040

[B2] Aru J. Aru J. Priesemann V. Wibral M. Lana L. Pipa G. . (2015). Untangling cross-frequency coupling in neuroscience. Curr. Opin. Neurobiol. 31, 51–61. doi: 10.1016/j.conb.2014.08.002 25212583

[B3] Battiston F. Cencetti G. Iacopini I. Latora V. Lucas M. Patania A. . (2020). Networks beyond pairwise interactions: structure and dynamics. Phys. Rep. 874, 1–92. doi: 10.1016/j.physrep.2020.05.004

[B4] Birren J. E. Fisher L. M. (1995). Aging and speed of behavior: Possible consequences for psychological functioning. Annu. Rev. Psychol. 46:329. doi: 10.1146/annurev.ps.46.020195.001553 7872732

[B5] Cesnaite E. Steinfath P. Idaji M. J. Stephani T. Kumral D. Haufe S. . (2023). Alterations in rhythmic and non-rhythmic resting-state EEG activity and their link to cognition in older age. Neuroimage 268:119810. doi: 10.1016/j.neuroimage.2022.119810 36587708

[B6] Chiang A. Rennie C. Robinson P. Van Albada S. Kerr C. (2011). Age trends and sex differences of alpha rhythms including split alpha peaks. Clin. Neurophysiol. 122, 1505–1517. doi: 10.1016/j.clinph.2011.01.040 21349761

[B7] Dimitriadis S. Laskaris N. Micheloyannis S. (2015). Transition dynamics of EEG-based network microstates during mental arithmetic and resting wakefulness reflects task-related modulations and developmental changes. Cogn. Neurodyn. 9, 371–387. doi: 10.1007/s11571-015-9330-8 26157511 PMC4491334

[B8] Dimitriadis S. Laskaris N. Tzelepi A. (2013). On the quantization of time-varying phase synchrony patterns into distinct functional connectivity microstates (fcμstates) in a multi-trial visual erp paradigm. Brain Topogr. 26, 397–409. doi: 10.1007/s10548-013-0276-z 23443252

[B9] Esghaei M. Treue S. Vidyasagar T. R. (2022). Dynamic coupling of oscillatory neural activity and its roles in visual attention. Trends Neurosci. 45, 323–335. doi: 10.1016/j.tins.2022.01.003 35190202

[B10] Gray R. T. Robinson P. A. (2013). Stability constraints on large-scale structural brain networks. Front. Comput. Neurosci. 7:31. doi: 10.3389/fncom.2013.00031 23630490 PMC3624092

[B11] Guan K. Zhang Z. Chai X. Tian Z. Liu T. Niu H. (2022). Eeg based dynamic functional connectivity analysis in mental workload tasks with different types of information. IEEE Trans. Neural Syst. Rehabilit. Eng. 30, 632–642. doi: 10.1109/TNSRE.2022.3156546 35239485

[B12] Hao Z. Zhai X. Cheng D. Pan Y. Dou W. (2022). Eeg microstate-specific functional connectivity and stroke-related alterations in brain dynamics. Front. Neurosci. 16:848737. doi: 10.3389/fnins.2022.848737 35645720 PMC9131012

[B13] Hutchison R. M. Womelsdorf T. Allen E. A. Bandettini P. A. Calhoun V. D. Corbetta M. . (2013). Dynamic functional connectivity: promise, issues, and interpretations. Neuroimage 80, 360–378. doi: 10.1016/j.neuroimage.2013.05.079 23707587 PMC3807588

[B14] Khanna A. Pascual-Leone A. Michel C. M. Farzan F. (2015). Microstates in resting-state EEG: current status and future directions. Neurosci. Biobehav. Rev. 49, 105–113. doi: 10.1016/j.neubiorev.2014.12.010 25526823 PMC4305485

[B15] Lehmann D. (1971). Multichannel topography of human alpha EEG fields. Electroencephalogr. Clin. Neurophysiol. 31, 439–449. doi: 10.1016/0013-4694(71)90165-9 4107798

[B16] Lehmann D. Ozaki H. Pál I. (1987). Eeg alpha map series: brain micro-states by space-oriented adaptive segmentation. Electroencephalogr. Clin. Neurophysiol. 67, 271–288. doi: 10.1016/0013-4694(87)90025-3 2441961

[B17] Merkin A. Sghirripa S. Graetz L. Smith A. E. Hordacre B. Harris R. . (2023). Do age-related differences in aperiodic neural activity explain differences in resting EEG alpha? Neurobiol. Aging 121, 78–87. doi: 10.1016/j.neurobiolaging.2022.09.003 36379095

[B18] Michel C. M. Koenig T. (2018). Eeg microstates as a tool for studying the temporal dynamics of whole-brain neuronal networks: a review. Neuroimage 180, 577–593. doi: 10.1016/j.neuroimage.2017.11.062 29196270

[B19] Musso F. Brinkmeyer J. Mobascher A. Warbrick T. Winterer G. (2010). Spontaneous brain activity and EEG microstates. A novel EEG/fmri analysis approach to explore resting-state networks. Neuroimage 52, 1149–1161. doi: 10.1016/j.neuroimage.2010.01.093 20139014

[B20] Nava E. Giraud M. Bolognini N. (2024). The emergence of the multisensory brain: From the womb to the first steps. Iscience 27:108758. doi: 10.1016/j.isci.2023.108758 38230260 PMC10790096

[B21] Nobukawa S. Ikeda T. Kikuchi M. Takahashi T. (2021). “Dynamical characteristics of state transition defined by neural activity of phase in Alzheimer's disease,” in International Conference on Neural Information Processing (Springer), 46–54. doi: 10.1007/978-3-030-92310-5_6

[B22] Nobukawa S. Ikeda T. Kikuchi M. Takahashi T. (2024). Atypical instantaneous spatio-temporal patterns of neural dynamics in Alzheimer's disease. Sci. Rep. 14:88. doi: 10.1038/s41598-023-50265-3 38167950 PMC10761722

[B23] Nobukawa S. Kikuchi M. Takahashi T. (2019). Changes in functional connectivity dynamics with aging: a dynamical phase synchronization approach. Neuroimage 188, 357–368. doi: 10.1016/j.neuroimage.2018.12.008 30529509

[B24] O'Connor S. Robinson P. (2004). Spatially uniform and nonuniform analyses of electroencephalographic dynamics, with application to the topography of the alpha rhythm. Phys. Rev. E–Stat. Nonl. Soft Matter Phys. 70:011911. doi: 10.1103/PhysRevE.70.011911 15324092

[B25] Rajkumar R. Farrher E. Mauler J. Sripad P. Régio Brambilla C. Rota Kops E. . (2021). Comparison of EEG microstates with resting state fmri and fdg-pet measures in the default mode network via simultaneously recorded trimodal (pet/mr/EEG) data. Hum. Brain Mapp. 42, 4122–4133. doi: 10.1002/hbm.24429 30367727 PMC8356993

[B26] Raut R. V. Snyder A. Z. Mitra A. Yellin D. Fujii N. Malach R. . (2021). Global waves synchronize the brain's functional systems with fluctuating arousal. Sci. Adv. 7:eabf2709. doi: 10.1126/sciadv.abf2709 34290088 PMC8294763

[B27] Robinson P. Loxley P. O'connor S. Rennie C. (2001). Modal analysis of corticothalamic dynamics, electroencephalographic spectra, and evoked potentials. Phys. Rev. E 63:041909. doi: 10.1103/PhysRevE.63.041909 11308879

[B28] Robinson P. Whitehouse R. Rennie C. (2003). Nonuniform corticothalamic continuum model of electroencephalographic spectra with application to split-alpha peaks. Phys. Rev. E 68:021922. doi: 10.1103/PhysRevE.68.021922 14525021

[B29] Sporns O. Betzel R. F. (2016). Modular brain networks. Annu. Rev. Psychol. 67, 613–640. doi: 10.1146/annurev-psych-122414-033634 26393868 PMC4782188

[B30] Thiebaut de Schotten M. Forkel S. J. (2022). The emergent properties of the connected brain. Science 378, 505–510. doi: 10.1126/science.abq2591 36378968

[B31] Tobe M. Nobukawa S. Mizukami K. Kawaguchi M. Higashima M. Tanaka Y. . (2023). Hub structure in functional network of EEG signals supporting high cognitive functions in older individuals. Front. Aging Neurosci. 15:1130428. doi: 10.3389/fnagi.2023.1130428 37139091 PMC10149684

[B32] Van de Ville D. Britz J. Michel C. M. (2010). “EEG microstate sequences in healthy humans at rest reveal scale-free dynamics,” in Proceedings of the National Academy of Sciences, 201007841. doi: 10.1073/pnas.1007841107 20921381 PMC2964192

[B33] Yan T. Wang G. Liu T. Li G. Wang C. Funahashi S. . (2023). Effects of microstate dynamic brain network disruption in different stages of schizophrenia. IEEE Trans. Neural Syst. Rehabilit. Eng. 31, 2688–2697. doi: 10.1109/TNSRE.2023.3283708 37285242

[B34] Zhang K. Shi W. Wang C. Li Y. Liu Z. Liu T. . (2021). Reliability of EEG microstate analysis at different electrode densities during propofol-induced transitions of brain states. Neuroimage 231:117861. doi: 10.1016/j.neuroimage.2021.117861 33592245

