# Correction: Age-group differences between young and middle-aged adults in spatiotemporal EEG dynamics revealed by instantaneous frequency microstate analysis

**DOI:** 10.3389/fnagi.2026.1875647

**Published:** 2026-05-21

**Authors:** Sou Nobukawa, Takashi Ikeda, Mitsuru Kikuchi, Tetsuya Takahashi

**Affiliations:** 1 Department of Computer Science, Chiba Institute of Technology, Narashino, Chiba, Japan; 2 Graduate School of Information and Computer Science, Chiba Institute of Technology, Narashino, Chiba, Japan; 3 Research Center for Mathematical Engineering, Chiba Institute of Technology, Narashino, Chiba, Japan; 4 Department of Preventive Intervention for Psychiatric Disorders, National Institute of Mental Health, National Center of Neurology and Psychiatry, Tokyo, Japan; 5 Research Center for Child Mental Development, Kanazawa University, Kanazawa, Ishikawa, Japan; 6 Department of Psychiatry and Behavioral Science, Kanazawa University, Kanazawa, Ishikawa, Japan; 7 Department of Neuropsychiatry, University of Fukui, Yoshida, Fukui, Japan; 8 Uozu Shinkei Sanatorium, Uozu, Toyama, Japan

**Keywords:** aging, aperiodic activity, electroencephalography (EEG), hidden Markov model (HMM), instantaneous frequency (IF), microstates

In the published article, there was an error in the **Funding** statement. Grant number JP25K00148 was erroneously omitted from the JSPS KAKENHI Grant-in-Aid for Scientific Research (B) funding information. The correct **Funding** statement appears below:

**Funding**

The author(s) declared that financial support was received for this work and/or its publication. This work was supported by JSPS KAKENHI: Grant-in-Aid for Scientific Research (C) [grant numbers JP22K12183 (SN) and JP23K06983 (TT)], Grant-in-Aid for Scientific Research (B) [grant numbers JP25K03198 and JP25K00148 (SN)], and Grant-in-Aid for Transformative Research Areas (A) [grant number JP25H02626 (SN)]. This work was partially supported by Project JPNP14004, commissioned by the New Energy and Industrial Technology Development Organization (NEDO).

The original version of this article has been updated.

