# Supplementary material for: Age-group differences between young and middle-aged adults in spatiotemporal EEG dynamics revealed by instantaneous frequency microstate analysis
Source: Front Aging Neurosci. 2026 Mar 26;18:1707228. doi: 10.3389/fnagi.2026.1707228 (PMC13061876; doi:10.3389/fnagi.2026.1707228)
Supplement: Supplementary file 1 [file Data_Sheet_1.pdf]

Supplementary Material: Age-group differences between young and middle-aged adults in spatiotemporal EEG dynamics revealed by instantaneous frequency microstate analysis

1 INSTANTANEOUS FREQUENCY MICROSTATE ANALYSIS IN THE ALPHA BAND

In the main analysis, instantaneous frequency (IF) microstates were derived from EEG signals band-pass filtered in the theta − alpha range (4-13 Hz), following our previous study Nobukawa et al. (2024). To further examine the robustness of the IF microstate structure under a narrower frequency condition, we additionally performed IF microstate analysis restricted to the alpha band (8-13 Hz), which is the dominant oscillatory component during the eyes-closed resting state. The EEG signals were band-pass filtered within the alpha range prior to application of the Hilbert transform. The analytic signal was then computed from this band-limited oscillatory process, from which IF was estimated. Figure S1 (A) shows the group-averaged spatial distributions of z-scored IF for each IF microstate. Consistent with the main analysis using the 4-13 Hz band, spatial patterns characterized by relatively faster occipital IF were clearly observed in several microstates (e.g., #3, #4, and #5). Furthermore, the group comparisons of mean dwell time and occupancy (Fig. S1 (B)) revealed significant differences between the younger and middle-aged groups (false discovery rate (FDR) corrected, $q < 0.05$), consistent with the results obtained in the broader 4-13 Hz band. These results indicate that the IF microstate organization and group differences are preserved when the analysis is restricted to the alpha band, supporting the robustness of the main findings.

REFERENCES

Nobukawa S, Ikeda T, Kikuchi M, Takahashi T. Atypical instantaneous spatio-temporal patterns of neural dynamics in Alzheimer's disease. *Scientific Reports* **14** (2024) 88.

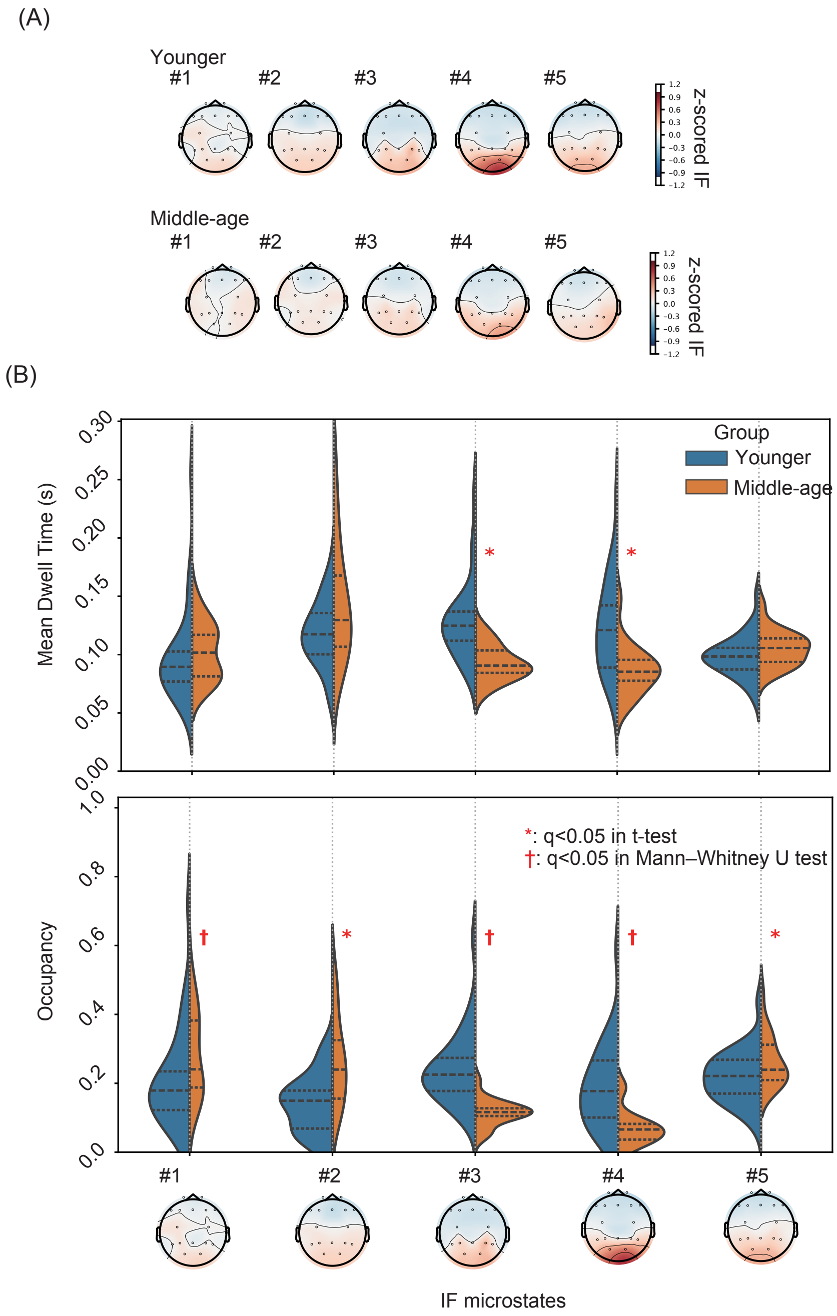

Figure S1. (A) Group-averaged spatial distributions of z-scored IF for each IF microstate in the younger and middle-aged groups, estimated from alpha-band (8-13 Hz) EEG signals. A pattern characterized by relatively faster occipital IF is observed in several microstates (e.g., #3, #4, and #5). (B) Violin plots of mean dwell time (top) and occupancy (bottom) for each IF microstate in the younger and middle-aged groups. The black solid and dashed lines indicate the group mean and standard deviation, respectively. Significant differences between groups (false discovery rate (FDR) corrected, $q < 0.05$) were observed, consistent with the analysis using the 4-13 Hz band.
